# Supplementary material for: Squidly harnesses enzyme functional hierarchy and contrastive learning to efficiently predict catalytic residues from sequence
Source: eLife. 2026 Sep 4;14:RP108186. doi: 10.7554/eLife.108186 (PMC13544906; doi:10.7554/eLife.108186)
Supplement: Peer review history

# Editor report: eLife Assessment

**DOI:** 10.7554/eLife.108186.3.sa0

**Authors:** Martin Graña

**Affiliations:** Institut Pasteur de Montevideo Uruguay

**Keywords:** Convincing, Important

This **important** contribution to enzyme annotation offers a deep learning framework for catalytic site prediction. Integrating biochemical knowledge with large language models, the authors demonstrate how to extract meaningful information from sequence alone. They introduce Squidly, a freely available new ML modeling framework, that outperforms existing tools on standard benchmarks, including the CataloDB dataset. The evidence is **convincing**, with an extensively and carefully addressed narrative upon revision.

# Referee report: Reviewer #1 (Public review)

**DOI:** 10.7554/eLife.108186.3.sa1

In this well-written and timely manuscript, Rieger et al. introduce Squidly, a new deep learning framework for catalytic residue prediction. The novelty of the work lies in the aspect of integrating per-residue embeddings from large protein language models (ESM2) with a biology-informed contrastive learning scheme that leverages enzyme class information to rationally mine hard positive/negative pairs. Importantly, the method avoids reliance on the use of predicted 3D structures, enabling scalability, speed, and broad applicability. The authors show that Squidly outperforms existing ML-based tools and even BLAST in certain settings, while an ensemble with BLAST achieves state-of-the-art performance across multiple benchmarks. Additionally, the introduction of the CataloDB benchmark, designed to test generalization at low sequence and structural identity, represents another important contribution of this work.

# Referee report: Reviewer #2 (Public review)

**DOI:** 10.7554/eLife.108186.3.sa2

Summary:

The authors aim to develop Squidly, a sequence-only catalytic residue prediction method. By combining protein language model (ESM2) embedding with a biologically inspired contrastive learning pairing strategy, they achieve efficient and scalable predictions without relying on three-dimensional structure. Overall, the authors largely achieved their stated objectives, and the results generally support their conclusions. This research has the potential to advance the fields of enzyme functional annotation and protein design, particularly in the context of screening large-scale sequence databases and unstructured data. However, the data and methods are still limited by the biases of current public databases, so the interpretation of predictions requires specific biological context and experimental validation.

Strengths:

The strengths of this work include the innovative methodological incorporation of EC classification information for "reaction-informed" sample pairing, thereby enhancing the discriminative power of contrastive learning. Results demonstrate that Squidly outperforms existing machine learning methods on multiple benchmarks and is significantly faster than structure prediction tools, demonstrating its practicality.

# Author comment: Author response

**DOI:** 10.7554/eLife.108186.3.sa3

**Authors:** William JF Rieger, Mikael Bodén, Frances Arnold, Ariane Mora

**Affiliations:** The University of Queensland Brisbane Australia; The University of Queensland Brisbane Australia; California Institute of Technology Pasadena United States; California Institute of Technology Pasadena United States

The following is the authors’ response to the original reviews.

**Reviewer #1:**

In this well-written and timely manuscript, Rieger et al. introduce Squidly, a new deep learning framework for catalytic residue prediction. The novelty of the work lies in the aspect of integrating per-residue embeddings from large protein language models (ESM2) with a biology-informed contrastive learning scheme that leverages enzyme class information to rationally mine hard positive/negative pairs. Importantly, the method avoids reliance on the use of predicted 3D structures, enabling scalability, speed, and broad applicability. The authors show that Squidly outperforms existing ML-based tools and even BLAST in certain settings, while an ensemble with BLAST achieves state-of-the-art performance across multiple benchmarks. Additionally, the introduction of the CataloDB benchmark, designed to test generalization at low sequence and structural identity, represents another important contribution of this work.

We thank the reviewer for their constructive and encouraging assessment of the manuscript. We appreciate the recognition of Squidly’s biology-informed contrastive learning framework with ESM2 embeddings, its scalability through the avoidance of predicted 3D structures, and the contribution of the CataloDB benchmark. We are pleased that the reviewer finds these aspects to be of value, and their comments will help us in further clarifying the strengths and scope of the work.

The manuscript acknowledges biases in EC class representation, particularly the enrichment for hydrolases. While CataloDB addresses some of these issues, the strong imbalance across enzyme classes may still limit conclusions about generalization. Could the authors provide per-class performance metrics, especially for underrepresented EC classes?

We thank the reviewer for raising this point. We agree that per-class performance metrics provide important insight into generalizability across underrepresented EC classes. In response, we have updated Figure 3 to include two additional panels: (i) per-EC F1, precision and recall scores, and (ii) a relative display of true positives against the total number of predictable catalytic residues. These additions allow the class imbalance to be more directly interpretable. We have also revised the text between lines 316-321 to better contextualize our generalizability claims in light of these results.

An ablation analysis would be valuable to demonstrate how specific design choices in the algorithm contribute to capturing catalytic residue patterns in enzymes.

We agree an ablation analysis is beneficial to show the benefits of a specific approach. We consider the main design choice in Squidly to be how we select the training pairs, hence we chose a standard design choice for the contrastive learning model. We tested the effect of different pair schemes on performance and report the results in Figure 2A and lines 244258. These results are a targeted ablation in which we evaluate Squidly against AEGAN using the AEGAN training and test datasets, while systematically varying the ESM2 model size and pair-mining scheme. As a baseline, we included the LSTM trained directly on ESM2 embeddings and random pair selection. We showed that indeed the choice of pairs has a large impact on performance, which is significantly improved when compared to naïve pairing. This comparison suggests that performance gains are attributable to reactioninformed pair-mining strategies. We recognize that the way these results were originally presented made this ablation less clear. We have revised the wording in the Results section (lines 244-247) and updated the caption to Figure 2A to emphasize the purpose of this section of the paper.

The statement that users can optionally use uncertainty to filter predictions is promising but underdeveloped. How should predictive entropy values be interpreted in practice? Is there an empirical threshold that separates high- from low-confidence predictions? A demonstration of how uncertainty filtering shifts the trade-off between false positives and false negatives would clarify the practical utility of this feature.

Thank you for the suggestion. Your comment prompted us to consider what is the best way to represent the uncertainty and, additionally, what is the best metric to return to users and how to visualize the results. Based on this, we included several new figures (Figure 3H and Supplementary Figures S3-5). We used these figures to select the cutoffs (mean prediction of 0.6, and variance < 0.225) which were then set as the defaults in Squidly, and used in all subsequent analyses. The effect of these cutoffs is most evident in the tradeoff of precision and recall. Hence users may opt to select their own filters based on the mean prediction and variance across the predictions, and these cutoffs can be passed as command line parameters to Squidly. The choice to use a consistent default cutoff selected using the Uni3175 benchmark has slightly improved the reported performance for the benchmarks seen in table 1, and figure 3C. However, our interpretation remains the same.

The excerpt highlights computational efficiency, reporting substantial runtime improvements (e.g., 108 s vs. 5757 s). However, the comparison lacks details on dataset size, hardware/software environment, and reproducibility conditions. Without these details, the speedup claim is difficult to evaluate. Furthermore, it remains unclear whether the reported efficiency gains come at the expense of predictive performance

Thank you for pointing out this limitation in how we presented the runtime results. We have rerun the tests and updated the table. An additional comment is added underneath, which details the hardware/software environment used to run both tools, as well as that the Squidly model is the ensemble version. As per the relationship between efficiency gains and predictive performance, both 3B and 15B models are benchmarked side by side across the paper.

Compared to the tools we were able to comprehensively benchmark, it does not come at a cost. However, we note that the increased benefits in runtime assume that a structure must be folded, which is not the case for enzymes already present in the PDB. If that is the case, then it is likely already annotated and, in those cases, we recommend using BLAST which is superior in terms of run time than either Squidly or a structure-based tool and highly accurate for homologous or annotated sequences.

Given the well-known biases in public enzyme databases, the dataset is likely enriched for model organisms (e.g., *E. coli*, yeast, human enzymes) and underrepresents enzymes from archaea, extremophiles, and diverse microbial taxa. Would this limit conclusions about Squidly's generalizability to less-studied lineages?

The enrichment for model organisms in public enzyme databases may indeed affect both ESM2 and Squidly when applied to underrepresented lineages such as archaea, extremophiles, and diverse microbial taxa. We agree that this limitation is significant and have adjusted and expanded the previous discussion of benchmarking limitations accordingly (lines 358, 369). We thank the reviewer for highlighting this issue, which has helped us to improve the transparency and balance of the manuscript.

**Reviewer #2:**

The authors aim to develop Squidly, a sequence-only catalytic residue prediction method. By combining protein language model (ESM2) embedding with a biologically inspired contrastive learning pairing strategy, they achieve efficient and scalable predictions without relying on three-dimensional structure. Overall, the authors largely achieved their stated objectives, and the results generally support their conclusions. This research has the potential to advance the fields of enzyme functional annotation and protein design, particularly in the context of screening large-scale sequence databases and unstructured data. However, the data and methods are still limited by the biases of current public databases, so the interpretation of predictions requires specific biological context and experimental validation.

Strengths:

The strengths of this work include the innovative methodological incorporation of EC classification information for "reaction-informed" sample pairing, thereby enhancing the discriminative power of contrastive learning. Results demonstrate that Squidly outperforms existing machine learning methods on multiple benchmarks and is significantly faster than structure prediction tools, demonstrating its practicality.

Weaknesses:

Disadvantages include the lack of a systematic evaluation of the impact of each strategy on model performance. Furthermore, some analyses, such as PCA visualization, exhibit low explained variance, which undermines the strength of the conclusions.

We thank the reviewer for their comments and feedback.

The authors state that "Notably, the multiclass classification objective and benchmarks used to evaluate EasIFA made it infeasible to compare performance for the binary catalytic residue prediction task." However, EasIFA has also released a model specifically for binary catalytic site classification. The authors should include EasIFA in their comparisons in order to provide a more comprehensive evaluation of Squidly's performance.

We thank the reviewer for raising this point. EasIFA’s binary classification task includes catalytic, binding, and “other” residues, which differs from Squidly’s strict catalytic residue prediction. This makes direct comparison non-trivial, which is why we originally had opted to not benchmark against EasIFA and instead highlight it in our discussion.

Given your comment, we did our best to include a benchmark that could give an indication of a comparison between the two tools. To do this, we filtered EasIFA’s multiclass classification test dataset for a non-overlapping subset with Squidly and AEGAN training data and <40% sequence identity to all training sets. This left only 66 catalytic residue– containing sequences that we could use as a held-out test set from both tools. We note it is not directly equal as Squidly and AEGAN had lower average identity to this subset (8.2%) than EasIFA (23.8%), placing them at a relative disadvantage.

We also identified a potential limitation in EasIFA’s original recall calculation, where sequences lacking catalytic residues were assigned a recall of 0. We adapted this to instead consider only the sequences which do have catalytic residues, which increased recall across all models. With the updated evaluation, EasIFA continues to show strong performance, consistent with it being SOTA if structural inputs are available. Squidly remains competitive given it operates solely from sequence and has a lower sequence identity to this specific test set.

Due to the small and imbalanced benchmark size, differences in training data overlap, and differences in our analysis compared with the original EasIFA analysis, we present this comparison in a new section (A.4) of the supplementary information rather than in the main text. References to this section have been added in the manuscript at lines 265-268. Additionally, we do update the discussion and emphasize the potential benefits of using EasIFA at lines (353-356).

The manuscript proposes three schemes for constructing positive and negative sample pairs to reduce dataset size and accelerate training, with Schemes 2 and 3 guided by reaction information (EC numbers) and residue identity. However, two issues remain:

(a) The authors do not systematically evaluate the impact of each scheme on model performance.

(b) In the benchmarking results, it is not explicitly stated which scheme was used for comparison with other models (e.g., Table 1, Figure 6, Figure 8). This lack of clarity makes it difficult to interpret the results and assess reproducibility.

(c) Regarding the negative samples in Scheme 3 in Figure 1, no sampling patterns are shown for residue pairs with the same amino acid, different EC numbers, and both being catalytic residues.

We thank the reviewer for these suggestions, which enabled us to improve the clarity and presentation of the manuscript. Please find our point by point response:

(a) We thank the reviewer for highlighting the lack of clarity in the way we have presented our evaluation in the section describing the Uni3175 benchmark. We aimed to systematically evaluate the impact of each scheme using the Uni3175 benchmark and refer to these results at lines 244-258, Additionally, we have adjusted the presentation of this section at lines 244-247 also in line with related comments from reviewer 1 in order to make the intention of this section and benchmark results to allow a comparison of each scheme to baseline models and AEGAN. These results led us to use Scheme 3 in both models for the other benchmarks in Figures 2 and 3. Please let us know if there is anything we can do to further improve the interpretability of Squidly’s performance.

(b) We thank the reviewer for highlighting this issue and improving the clarity of our manuscript. We agree that after the Uni3175 benchmark was used to evaluate the schemes, we did not clearly state in the other benchmarks that scheme 3 was chosen for both the 3B and 15B models. We have made changes in table 1 and the Figure legends of Figures 2 and 3 to state that scheme 3 was used. In addition, we integrated related results into panel figures (e.g. Figures 2 and 3 now show models trained and tested on consistent benchmark datasets) and standardized figure colors and legend formatting throughout. Furthermore, we suspect that the previous switch from using the individual vs ensembled Squidly models during the paper was not well indicated, and likely to confuse the reader. Therefore, we decided to consistently report the ensembled Squidly models for all benchmarks except in the ablation study (Figure 2A). In line with this, we altered the overview Figure 1A, so that it is clearer that the default and intended version of Squidly is the ensemble.

(c) We appreciate the reviewer pointing this out. You’re correct, we explicitly did not sample the negatives described by the reviewer in scheme 3 as our focus was on the hard negatives that relate most to the binary objective. We do think this is a great idea and would be worth exploring further in future versions of Squidly, where we will be expanding the label space used for hard-negative sampling and including binding sites in our prediction. We have updated the discussion at lines 395-396 to highlight this potential direction.

The PCA visualization (Figure 3) explains very little variance (~5% + 1.8%), but its use to illustrate the separability of embedding and catalytic residues may overinterpret the meaning of the low-dimensional projection. We question whether this figure is appropriate for inclusion in the main text and suggest that it be moved to the Supporting Information.

We thank the reviewer for this suggestion. We had discussed this as well, and in the end decided to include it in the main manuscript. We agree that the explained variance is low. However, when we first saw the PCA we were surprised that there was any separation at all. This then prompted us to investigate further, so we kept it in the manuscript to be true to the scientific story. However, we do agree that our interpretation could be interpreted as overly conclusive given the minimal variance explained by the top 2 PCs. Therefore, we agree with the assessment that the figure, alongside the accompanying results section, is more appropriately placed in the supplementary information. We moved this section (A.1) to the appendix to still explain the exploratory data analysis process that we used to tackle this problem, so that the general thought process behind Squidly is available for further reading.

Minor Comments:

(1) Figure Quality and Legends (a) In Figure 4, the legend is confusing: "Schemes 2 and 3 (S1 and S2) ..." appears inconsistent, and the reference to Scheme 3 (S3) is not clearly indicated.

(b) In Figure 6, the legend overlaps with the y-axis labels, reducing readability. The authors should revise the figures to improve clarity and ensure consistent notation.

The reviewer correctly notes inconsistencies in figure presentation. We have revised the legend of Figure 4 (now 2A) to ensure schemes are referred to consistently and Scheme 3 (S3) is clearly indicated. We also adjusted Figure 6 (now 2c) to remove the overlap between the legend and y-axis labels.

Conclusion

We thank the reviewers and editor again for their constructive input. We believe the revisions and clarifications substantially strengthened the manuscript and the resource
